# Therapy resistance in neuroblastoma: Mechanisms and reversal strategies

**DOI:** 10.3389/fphar.2023.1114295

**Published:** 2023-02-16

**Authors:** Xia Zhou, Xiaokang Wang, Nan Li, Yu Guo, Xiaolin Yang, Yuhe Lei

**Affiliations:** ^1^ Shenzhen Hospital of Guangzhou University of Chinese Medicine, Shenzhen, China; ^2^ Department of Pharmacy, Shenzhen Longhua District Central Hospital, Shenzhen, China; ^3^ Guangdong Provincial Key Laboratory of Research and Development of Natural Drugs, School of Pharmacy, Guangdong Medical University, Dongguan, China; ^4^ The Marine Biomedical Research Institute of Guangdong Zhanjiang, Zhanjiang, China; ^5^ School of Traditional Chinese Medicine, Jinan University, Guangzhou, China; ^6^ Shanghai University of Traditional Chinese Medicine, Shanghai, China

**Keywords:** neuroblastoma, therapy resistance, molecular mechanism, reversal strategy, cancer

## Abstract

Neuroblastoma is one of the most common pediatric solid tumors that threaten the health of children, accounting for about 15% of childhood cancer-related mortality in the United States. Currently, multiple therapies have been developed and applied in clinic to treat neuroblastoma including chemotherapy, radiotherapy, targeted therapy, and immunotherapy. However, the resistance to therapies is inevitable following long-term treatment, leading to treatment failure and cancer relapse. Hence, to understand the mechanisms of therapy resistance and discover reversal strategies have become an urgent task. Recent studies have demonstrated numerous genetic alterations and dysfunctional pathways related to neuroblastoma resistance. These molecular signatures may be potential targets to combat refractory neuroblastoma. A number of novel interventions for neuroblastoma patients have been developed based on these targets. In this review, we focus on the complicated mechanisms of therapy resistance and the potential targets such as ATP-binding cassette transporters, long non-coding RNAs, microRNAs, autophagy, cancer stem cells, and extracellular vesicles. On this basis, we summarized recent studies on the reversal strategies to overcome therapy resistance of neuroblastoma such as targeting ATP-binding cassette transporters, *MYCN* gene, cancer stem cells, hypoxia, and autophagy. This review aims to provide novel insight in how to improve the therapy efficacy against resistant neuroblastoma, which may shed light on the future directions that would enhance the treatment outcomes and prolong the survival of patients with neuroblastoma.

## 1 Introduction

Neuroblastoma (NB) is one of the most common pediatric solid tumors originated from the sympathetic nervous system. It accounts for 8%–10% of all pediatric cancers and approximately 15% of all childhood cancer-related deaths in the United States (Colon and Chung, 2011; Zafar et al., 2021). There are some racial differences in NB, with the disease being more common in those with European descent, and African-American children are inclined to represent higher-risk disease (Henderson T. O. et al., 2011b). The morbidity of NB ranks third in children with cancer, only second to leukemia and brain cancer. The incidence of NB was age-related, with an average age of 17.3 months at the time of clinical diagnosis, and 40% of children were diagnosed before 1 year of age (Whittle et al., 2017). NB has great differences in tissue distribution, clinical features, and pathological molecular biology. In terms of survival rate, 85%–90% of low-and intermediate-risk patients can be cured, while high-risk NB patients have very poor outcomes, with a 5-year survival rates below 50% (Pudela et al., 2020). About half of high-risk patients do not respond to first-line treatment options or relapse in 2 years. The prognosis of NB patients varies widely, depending on the age of the child, the tumor grade at diagnosis, and various molecular pathological features, especially the amplification of the MYCN oncogene (Whittle et al., 2017). Current therapies for NB include chemotherapy, radiotherapy, targeted therapy, and immunotherapy. The chemotherapeutic drugs used in the treatment of NB include vinca alkaloids, anthracyclines, epipodophyllotoxins, camptothecin, and others, all of which can cause apoptosis by destroying nucleotides or inhibiting mitosis (Pearson et al., 2008; Aktas et al., 2010). NB is sensitive to radiotherapy, and almost all children in the high-risk group need to receive radiotherapy for the tumor bed after intense chemotherapy (Matthay et al., 2012). The current targeted therapies for NB include targeting genetic aberrations, targeting disrupted signaling molecules, targeting norepinephrine and somatostatin receptors by radiopharmaceutical (Zafar et al., 2021). Immunotherapy, which can improve survival and quality of NB patients by reducing exposure to cytotoxic drugs, has been incorporated into first-line treatment protocols. Unfortunately, the therapy resistance often leads to treatment failure, manifested as tumor growth or recurrence, which is responsible for increasing cancer-related mortality (Li Y et al., 2017). Various signaling molecules or pathways were confirmed to participate in the initiation of therapy resistance of cancer, but there is a lack of review to summarize recent progress on the mechanism study and reversal strategies of NB resistance. In this review, we focus on the mechanisms of therapy resistance in NB and discuss multiple approaches to reverse resistance with a view to discovering more possibilities for NB treatments.

## 2 Current research progress on NB

### 2.1 The mechanisms of NB occurrence and development

The neural crest originates from the embryonic ectoderm and develops from the neural tube after it is closed (Simões-Costa and Bronner, 2013). The differentiation of neural crest cells into a huge variety of cells contributes to the emergence of different anatomical structures due to epithelial-mesenchymal transformation (EMT), where cells lose polarity and gain less adhesion, which allows neural crest cells to stratify and migrate from the neural tube. These cells migrate along fixed paths to many remote parts of the embryo, where they eventually differentiate into a variety of different cell types, including melanocytes, craniofacial chondrocytes and bone, smooth muscle cells, peripheral neurons, and glial cells (Bronner and Simões-Costa, 2016). Many researchers believe that tumors originated from the neural crest may be prone to metastatic disease due to the innate ability of neural crest cells to self-renew and migrate (Gupta et al., 2005; Bailey et al., 2012). NB usually arises from the adrenal medulla or paravertebral sympathetic ganglia, with distinct masses in the chest, neck, pelvis, and/or abdomen, and occurs during the development and differentiation of neural crest cells into sympathetic nerve cells (Matthay et al., 2016; Kholodenko et al., 2018). NB has unique and diverse biological characteristics, including chromosomal instability, gene variation, stem cell, EMT, and epigenetic abnormalities (Fusco et al., 2018; Tonini and Capasso, 2020). Clinically, NB represents a wide range of phenotypes, ranging from spontaneous regression of disease to persistent treatment-refractory progression and death from high-risk metastatic disease. At the cellular level, the heterogeneous behavior of NBs may stem from the arrest and dysregulation of normal neural crest development (Tomolonis et al., 2018). Under genetic, epigenetic, or chemical stress, normal developmental pathways in the neural crest cells become dysregulated, leading to NB tumorigenesis (Louis and Shohet, 2015). The differentiation of sympathoadrenal precursors of the neural crest cells into sympathetic ganglia cells and adrenal chromaffin cells requires several factors, including overexpression of nerve growth factor (*NGF*) and *MYCN*, SRY-associated HMG-box gene 10 (*Sox10*), and mammalian Achaete-SCUTE homologs 1 (*MASH1*) induced through bone morphogenetic proteins (*BMPs*) (Kholodenko et al., 2018). Transformation of persistent resting progenitors into NB cells requires anaplastic lymphoma kinase (ALK) mutation and *MYCN* expansion (Kholodenko et al., 2018). Under the pressure of oncogenic stimuli such as *MYCN* aberrant expression, dysregulation of the signaling pathway in neural crest cell may generate highly malignant NB cancer stem cells (CSC) subsets (Olsen et al., 2017).

### 2.2 Current treatments for NB

**FIGURE 1:**
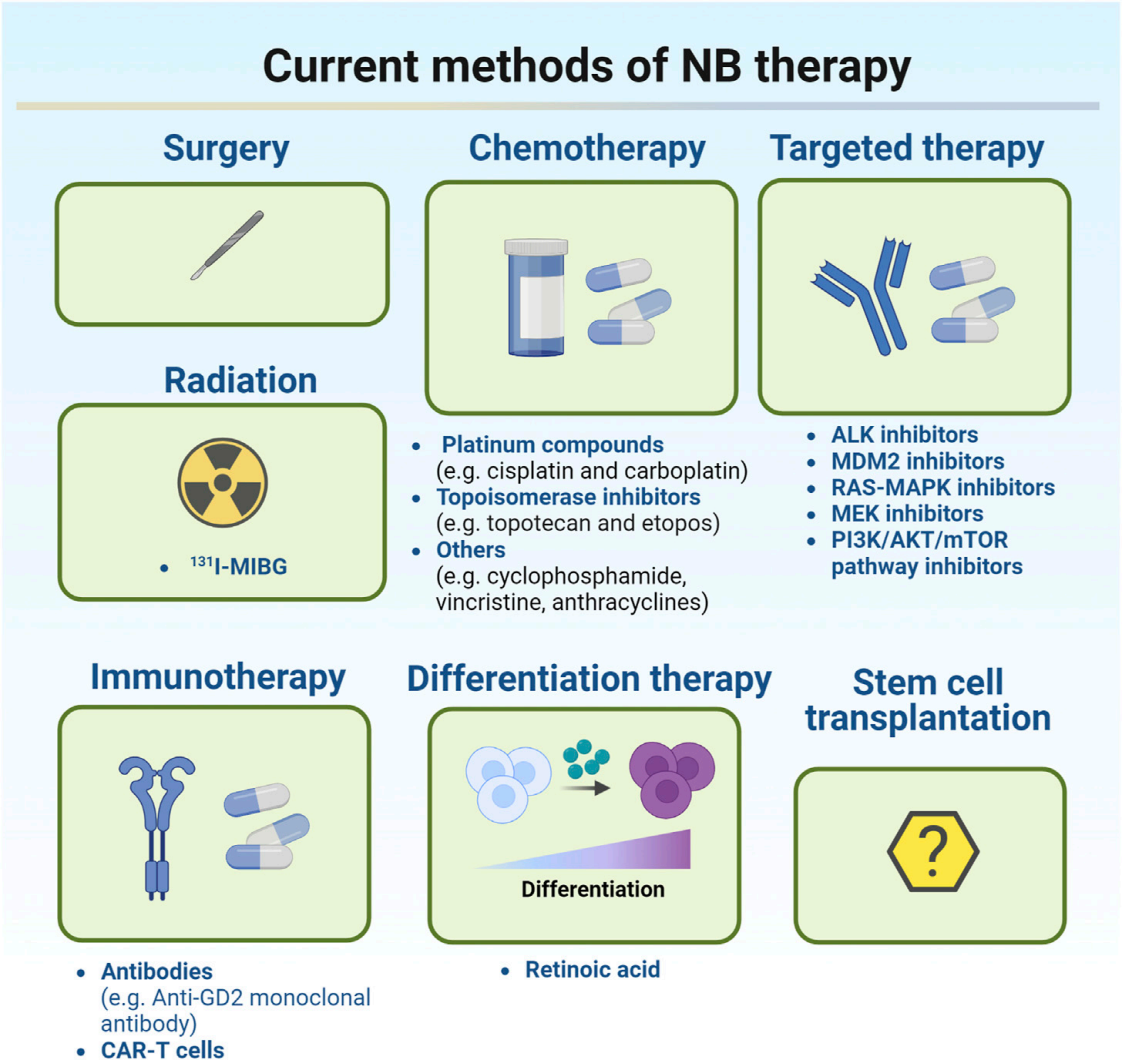
Current methods of NB therapy.

Current treatments for NB were illustrated in Figure 1.

Treatment of NB depends on the classification of the tumor. According to the degree of tumor cell differentiation, presence or absence of stroma, mitotic nuclear rupture index, patient age, histological type, *MYCN* oncogene status, DNA ploidy, and chromosome 11q status, NB patients were classified as very low risk, low risk, intermediate risk, or high risk, respectively (Valter et al., 2018). For very low-risk and low-risk NB, intensive treatment is not required because the tumors are likely to disappear on their own. For some smaller tumors which are generally easier to remove, surgery may be the best option for these patients. Chemotherapy can be applied after surgery, but is most commonly used to monitor patients for recurrence (Cohn et al., 2009). Common chemotherapy regimens such as carboplatin, cyclophosphamide, doxorubicin, and etoposide will be applied if the majority of the tumor cannot be removed surgically. Current chemotherapy approaches include induction chemotherapy and high-dose chemotherapy. The primary goal of induction chemotherapy is to reduce the initial metastatic tumor burden and subsequently increase the likelihood of successful surgery, stem cell transplantation, or other further treatments. Platinum compounds (cisplatin and carboplatin), topoisomerase inhibitors (topotecan and etoposide), and other chemotherapeutic compounds (cyclophosphamide, vincristine, and anthracyclines) are commonly applied in induction chemotherapy to induce a response (Park et al., 2011). Myeloablative high-dose chemotherapy has been reported to improve survival in patients with high-risk metastatic NB compared with non-myeloablative maintenance chemotherapy (Berthold et al., 2005). However, treatment with high-dose chemotherapy may increase the risk of acute and chronic organ toxicity. Radiation therapy is also an option if the patient does not respond adequately to chemotherapy. As a local treatment, radiotherapy has a clear role in improving the local control rate and alleviating symptoms such as tumor compression, bleeding, and pain in advanced children. Radiation therapies including external radiation and isotope radiation are the main strategies for the treatment of high-risk NB patient. ^131^ *I-MIBG* (methylphenylguanidine) therapy is an isotope radiotherapy targeting NB tissue. *MIBG* is a noradrenergic analog that targets 90% of the noradrenergic receptors expressed in NB cells. Currently, ^131^ *I-MIBG* therapy is commonly used for refractory and recurrent NB (Matthay et al., 2012). Although radiation therapy is recommended for nearly all high-risk NB patients, there is a lack of evidence to support its long-term application since its long-term side effects are already evident (Arumugam et al., 2019).

Currently, clinically available therapies for high-risk populations are multimodal treatments, including chemotherapy, stem cell transplantation, surgery, radiation therapy, retinoid therapy, and immunotherapy (Pezeshki et al., 2021). The current targeted therapies for NB include targeting genetic aberrations, targeting disrupted signaling molecules, immunology-based approaches, targeting norepinephrine and somatostatin receptors by radiopharmaceutical, targeting epigenetic modulators, and targeting Bcl-2 family proteins (Zafar et al., 2021). In recent years, the research on the molecular mechanism of NB pathogenesis has gradually increased. Ongoing studies have identified several signaling pathways required for NB growth and development, including the *PI3K/Akt/mTOR* pathway, p53-mouse double minute 2 homolog (*MDM2*) pathway (Lu et al., 2016), *RAS/MAPK* signaling pathway, and *ALK* signaling pathway (Infarinato et al., 2016), which may contribute to resistance of NB to conventional treatments. These aberrantly expressed genes and proteins may be the potential therapeutic targets for NB. Several inhibitors including *TRK*, *MYCN*, and *VEGF* inhibitors have been used in clinic (Muller et al., 2014; Iyer et al., 2016). Some clinical trials of small molecule inhibitors are under way. For example, *ALK* inhibitors, *MDM2* inhibitors, *RAS-MAPK* and *MEK* inhibitors, and *PI3K/AKT/mTOR* pathway inhibitors have been identified (Peirce et al., 2011; Kushner et al., 2017; Arumugam et al., 2019; Pezeshki et al., 2021). *ALK* inhibitors are clinically available and have been proved safe and effective in patients with recurrent and/or refractory NB (Qiu and Matthay, 2022). Effective immunotherapy can improve the survival rate and quality of NB patients by reducing exposure to cytotoxic agents. *GD2*, a surface glycolipid, is the most common target of immunotherapy (Sait and Modak, 2017; Jin et al., 2020). However, when anti-*GD2* monoclonal antibody was integrated into the standard upfront treatment regimen, the 5-year survival rate for NB patients is only about 50%. In the past few years, chimeric antigen receptor (CAR)-T cell therapy has shown its potential in the treatment of NB (Yan et al., 2019; Zhao and Cao, 2019). CAR-T cell therapy is to use the patient’s own T lymphocytes, transform them in the laboratory, load receptors and co-stimulatory molecules that can recognize tumor antigens, expand *in vitro*, and then reinject them into the patient’s body to recognize and attack its own tumor cells (Akhavan et al., 2019; Yadav et al., 2020). Currently, in terms of solid tumors, only NB patients have shown a good response to CAR-T cell therapy (Ma et al., 2019). The developed CAR-T cell surface targets for NB are *GD2, L1-CAM, GPC2, B7H3, ALK*, and *NCAM. ALK* CAR-T can target wild-type and mutant *ALK*, and CAR-T cells targeting GD2 and L1-CAM are currently in clinical trials (Richards et al., 2018). Differentiation therapy, as a new treatment method, has made some progress in the maintenance treatment of high-risk NB patients in recent years. The differentiation therapy involves reactivating the intrinsic differentiation program in cancer cells and forcing them to differentiate into mature, supposedly more benign cells (Jin et al., 2020). Retinoic acid is one of the differentiation modulators in cancer therapy (Brodeur and Bagatell, 2014). As a vitamin A derivative, retinoic acid has been shown to play a positive role in embryonic development, vision, metabolism, energy homeostasis, immune function, neural differentiation, and axonal growth (Janesick et al., 2015; Uray et al., 2016; de The, 2018). Retinoic acid can trigger differentiation by attaching to retinoic acid nuclear receptors, including retinoic acid receptors (RARs) and retinoic X receptors (RXRs). This complex in turn affects retinoic acid response elements, which are response genes regulating cell growth, differentiation, and apoptosis (Khalil et al., 2017). Studies have suggested that factors associated with retinoic acid uptake and storage by regulating intracellular retinoic acid may be targets for novel retinoic acid-based therapeutic strategies (Moise et al., 2007). At present, there is a lack of clinical studies on the stem cell transplantation in the treatment of recurrent and refractory NB in China. The existing small-scale studies demonstrated different efficacy of stem cell transplantation, which needs further clarification. If autologous stem cell collection meets the needs of transplantation, then the transplantation can be considered. For allogeneic hematopoietic stem cell transplantation (HSCT), unrelated ligand HSCT or hemiphase ligand HSCT can be considered according to the circumstances (Suh et al., 2020; Seo et al., 2022). At present, unified consensus has not been reached on the indications, timing selection, donor selection and preconditioning of HSCT in NB treatment, and more powerful data support needs to be provided by multi-center clinical studies with larger sample size.

## 3 Underlying mechanisms of therapy resistance in NB

**FIGURE 2:**
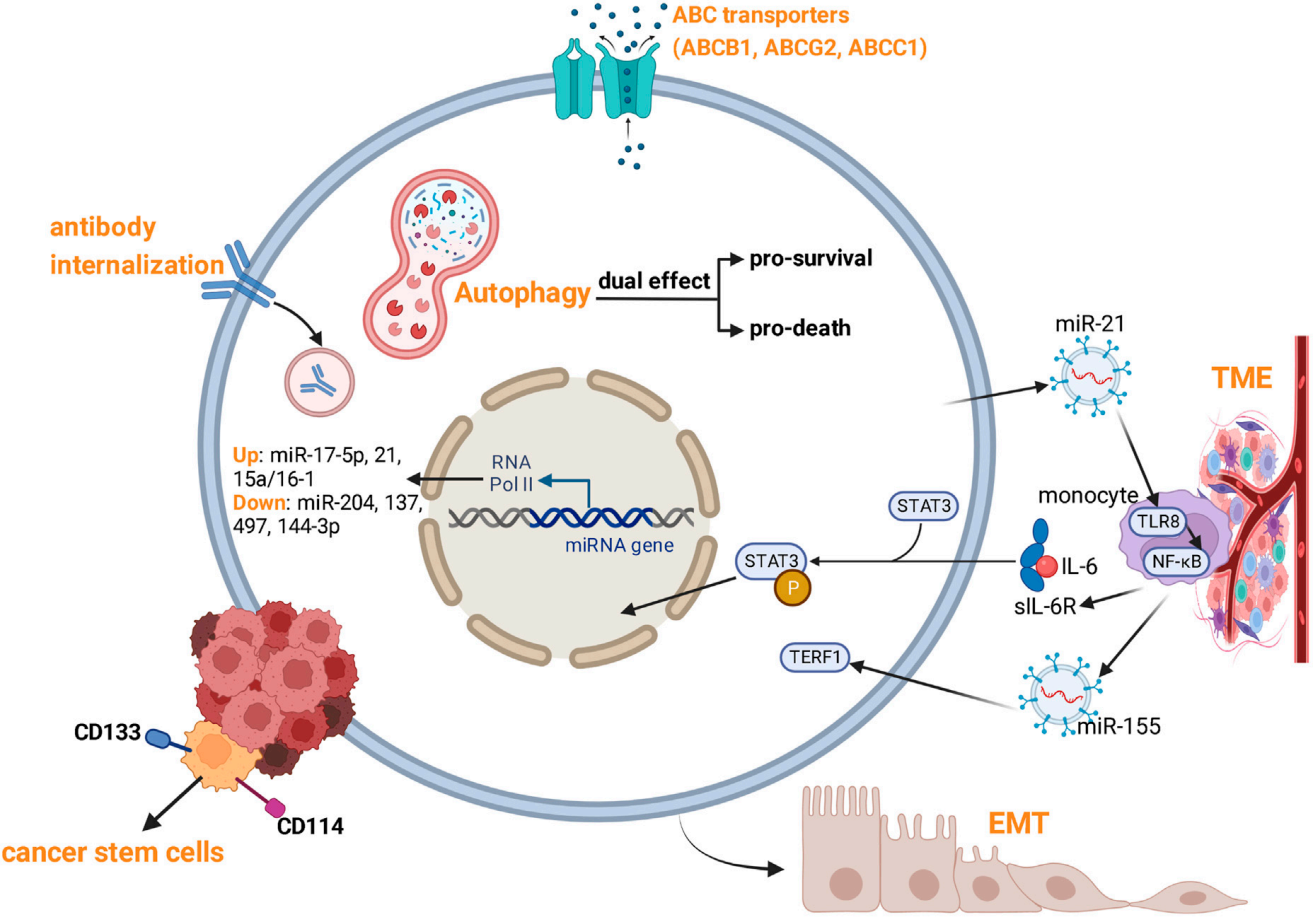
Mechanisms of therapy resistance of NB.

Despite significant medical advances in NB treatment, therapy resistance is a major barrier to access to curative cancer treatments. Hence, there is an urgent need to reveal the mechanisms of therapy resistance in NB. Cancer cells may either exhibit a significant primary resistance to drugs (primary resistance), or acquire characteristics of multi-drug resistance (MDR) after long-term chemotherapy (acquired resistance). NB exhibits inter- and intra-tumor genetic heterogeneity characterized by abnormal telomere maintenance mechanisms, *MYCN* amplification, and mutations in the RAS and/or p53 pathways, resulting in poor prognosis (Salemi et al., 2022). NB is composed of two epigenetically distinct cell types: undifferentiated mesenchymal cells (MES) and committed adrenergic cells (ADRN). MES cells expressing the stem cell marker CD133 are highly migratory and more resistant to chemotherapy, and are more common in tumors that recur after chemotherapy (Huang Y et al., 2021). Resistance of NB cells is usually caused by comprehensive mechanisms (Figure 2). In addition to genetic (mutation, amplification) and epigenetic changes (DNA hypermethylation, histone modifications), several mechanisms are involved: Overexpression of drug efflux transporters, aberrant expression of microRNAs (miRNAs), cancer cell stemness, autophagy, tumor microenvironment, extracellular vesicles, MEK/ERK signaling hyper-activation, and anti-disialoganglioside antibody internalization.

### 3.1 Drug export modulations mediated by the ATP-binding cassette genes

One of the most direct ways for tumors to become resistant to drug therapy is through physical mechanisms to block or restrict drug access to the site of action, one of which is by increasing the expression of ATP-binding cassette (ABC) transporter family proteins. An effective drug must be able to pass through the cell membrane and avoid being expelled out of the cell by efflux transporters. Overexpression of efflux transporters is associated with resistance to a large number of chemotherapeutic drugs, such as vinblastine, vincristine, doxorubicin, daunorubicin, and paclitaxel (Fletcher et al., 2016). The ABC transporter superfamily consists of 48 genes and is subdivided into 7 subfamilies, ranging from ABCA to ABCG (Dean, 2005b). Compelling evidence currently supports that three of these ATP-driven efflux transporters are responsible for chemoresistance *in vivo*, including *ABCB1* (P-glycoprotein (P-gp)/MDR1), *ABCG2* (Breast Cancer Resistance Protein/BCRP), and *ABCC1* (Multidrug Resistance Protein 1/MRP1) (Fletcher et al., 2016). Previous studies have demonstrated the direct contribution of MRP1 to tumor responsiveness to chemotherapy in a *MYCN* amplified NB (Burkhart et al., 2009). Many of the first-line drugs used in NB treatment are MRP1 substrates, including etoposide, doxorubicin, vincristine, and irinotecan. In addition to P-gp, BCRP, and MRP1, other ABC transporters are also capable to affect chemotherapy response *in vitro*, including several members of the ABCA and ABCB subfamilies and most members of the ABCC subfamily (Keppler, 2011). Some of the most compelling evidence for the participation of ABC transporters in cancer biology comes from NB. Several ABC transporters, including *ABCC1*, *ABCC3*, and *ABCC4*, are under direct transcriptional control of *MYCN*, and their expression is closely related to patient prognosis (Henderson M. J. et al., 2011a). Unlike *ABCC1* and *ABCC4*, *ABCC3* is negatively regulated by *MYCN* and its expression is usually very low in NB. *ABCC4* (multidrug resistance protein 4, MRP4) is transcriptionally regulated by *MYCN*, a driver of NB tumorigenesis and a recognized poor prognostic factor (Murray et al., 2017). High *ABCC4* mRNA expression strongly predicts poor clinical outcome (Henderson M. J. et al., 2011a). In cultured cells, *ABCC4* causes resistance to several anticancer drugs, including camptothecin and irinotecan (Bagatell et al., 2011).

### 3.2 Abnormal expression of microRNAs

Accumulating evidence indicated that microRNAs (miRNAs) play an important role in drug resistance in a variety of cancers (Giovannetti et al., 2012). MiRNAs are small (∼22 nucleotides) non-coding RNA molecules that regulate the expression of genes at the post-transcriptional level by either directly inhibiting the translation of target mRNA or accelerating its degradation (Chen and Hu, 2012). Genome-wide miRNA profiling revealed aberrant expressions in the majority of human cancers, suggesting an important role of miRNAs in carcinogenesis and tumor progression by acting as oncogenes or tumor suppressors (Kong et al., 2012). Several studies have shown that aberrant expression of certain miRNA is associated with poor clinical outcomes in NB (Buechner and Einvik, 2012), and the contribution of different miRNAs to NB resistance was also emphasized (Table 1). MiR-17–5p was found for the first time to be associated with drug resistance in NB. MiR-17–5p directly targets the tumor suppressor gene *P21* and the apoptotic *BIM* gene to promote NB cell proliferation and therapy resistance (Fontana et al., 2008; Galardi et al., 2018). MiR-204 binds to the 3′-untranslated region (UTR) of the anti-apoptotic gene *BCL2* and the oncogene *NTRK2*, both of which are associated with poor survival in drug-resistant NB patients (Ryan et al., 2012). This cluster represses the *p21* gene responsible for cell cycle progression and apoptosis, which subsequently renders them more resistant to chemotherapy in *MYCN*-amplified NB cells (Fontana et al., 2008). MiR-21 is upregulated in cisplatin-resistant SH-SY5Y and BE (2)-M17 NB cells compared with parental cells. In addition, ectopic expression of pre-miR-21 in parental cells resulted in increased drug resistance and proliferation rates since *PTEN* is the target of miR-21 (Chen et al., 2012; Buhagiar and Ayers, 2015). The expression of miR-137 and the constitutive androstane receptor (CAR) was negatively correlated in parental and doxorubicin-resistant NB cells, and miR-137 was downregulated in doxorubicin-resistant cells. Therefore, reinforcement of miR-137 suppressed CAR expression and re-sensitized NB cells to doxorubicin (Takwi et al., 2014). MiR-497 may regulate proliferation, survival, and tumor vascular permeability of chemoresistant NB cells possibly by targeting genes involved in DNA damage response (*WEE1* and *CHEK1*), cell growth and survival (*AKT3* and *BCL2*), and angiogenesis (*VEGFA*) (Soriano et al., 2016). In NB model cell lines, etoposide-resistance was associated with mono-allelic deletion of 13q14.3 and downregulation of miRNA-15a/16–1 (Marengo et al., 2018). Wang et al. demonstrated that non-coding RNA activated by DNA damage (NORAD) promoted the progression and DOX resistance of NB through miR-144–3p/HDAC8 axis *in vitro* and *in vivo* (Wang S. Y et al., 2020). In non-*MYCN* amplified NB cells, neural apoptosis inhibitory protein (NAIP) was reported to contribute to drug resistant phenotype by apoptosis inhibition. The increased NAIP level in NB cells resistant to cisplatin and etoposide partially resulted from the decreased level of miR-520f (Harvey et al., 2015). As a tumor suppressor, miR-34a also participates in the therapy resistance of progressive NB since it targets crucial players of therapy resistance including *N-MYC*, *E2F3*, *BCl2*, *CCND1*, and *CDK6* (Sun et al., 2008; Wei et al., 2008).

**TABLE 1:** miRNAs involved in drug resistance in NB.

Name	Drug	Sensitivity	Target	References
MiR-17–5p	Not specified	Down	*P21, BIM*	Fontana et al. (2008), Galardi et al. (2018)
MiR-204	Cisplatin, etoposide	Up	*BCL2, NTRK2*	Ryan et al. (2012)
MiR-21	Cisplatin	Down	*PTEN*	Fontana et al. (2008)
MiR-137	Doxorubicin	Up	*CAR, HDAC8*	Takwi et al. (2014)
MiR-497	Cisplatin, etoposide, melphalan	Up	*WEE1, CHEK1, AKT3, BCL2, VEGFA*	Soriano et al. (2016)
MiR-15a/16–1	Etoposide	Down	*P53*	Marengo et al. (2018)
MiR-144–3p	Doxorubicin	Up	*HDAC8*	Wang B et al. (2020)
MiR-520f	Cisplatin, etoposide	Up	*NAIP*	Harvey et al. (2015)
MiR-34a	Not specified	Down	*N-MYC, E2F3, BCl2, CCND1, and CDK6*	Sun et al. (2008), Wei et al. (2008)

*P21*, p21^Cip1/Waf1/Sdi1^; *BIM*, Bcl-2 interacting mediator of cell death; *BCL2*, B-cell Lymphoma 2; *NTRK2*, Neurotrophin Receptor Kinase 2; *PTEN*, Phosphatase Tensin Homologue; *CAR*, Constitutive Androstane Receptor; *HDAC8*, Histone Deacetilase 8; *WEE1*, a nuclear kinase belonging to the Ser/Thr family of protein kinases; *CHEK1*, Checkpoint Kinase 1; *AKT* 3, Serine/Threonine Kinase 3; *VEGFA*, Vascularendothelial growth factor A; *P53*, Tumor Protein 53; *NAIP*, Neural apoptosis inhibitory protein; *N-MYC*, a protein that in humans is encoded by the MYCN gene; *E2F3*, E2 F transcription factor 3; *CCND1*, cyclin D1; *CDK6*, cyclin-dependent kinase 6.

### 3.3 Involvement of cancer stem cells

Cancer stem cells (CSCs) are a small subpopulation of cancer cells with stem cell-like characteristics such as self-renewal and multi-directional differentiation ability (Yang et al., 2020). Stem cells come from three sources: the inner cell mass of embryos, induced pluripotent from normal somatic cells, and somatic adult stem cells (Takahashi et al., 2007). In 1994, CSCs were discovered in leukemia, and since then, they have been found in other solid tumors (Lapidot et al., 1994). Accumulating evidence suggests that CSCs play a key role in tumorigenesis, progression, metastasis, and recurrence (Veschi et al., 2019). These CSCs have the ability to generate cells that differentiate into tumor conditions with multidrug resistance properties (Borst, 2012). The high-risk NBs consist of small populations of cells with retained stem cell characteristics. These clones show the ability to form highly resistant tumorspheres with high metastasis potential (Hansford et al., 2007). The presence, clonal selection, and enrichment of CSCs contribute to NB progression, resistance to therapeutic measures, and poor prognosis. This function is caused by a variety of mechanisms, including inhibition of apoptosis, increased repair of DNA damage, conservation of dormancy, and altered drug response (Dean, 2005a). Due to the resistance of CSCs to chemotherapy, many scholars believe that CSCs are the main cause of NB recurrence and poor survival rate (Hansford et al., 2007). Discriminating the surface expression of select CSC markers clearly provides the basis of the CSC composition in NB as such and for drug reaction. Identification of specific surface markers facilitates characterization of CSCs, examination of NB biology/evolution and therapeutic targeting (Aravindan et al., 2019). So far, many cell surface CSC markers, including *CD133*, frizzled class receptor 6 (*FZD6*), leucine rich repeat containing GPCR 5 (*LGR5*), aldehyde dehydrogenase (*ALDH*), *ALDH1A2*, *ALDH1A3*, cluster of differentiation 114 (*CD114*), and cluster of differentiation 117 (*C-kit*), have been identified in NB (Shohet et al., 2011). These markers were confirmed to play a role in the initiation of NB therapy resistance. CD133 (Promin-1) is a transmembrane protein expressed in neural stem cells and has been shown to be a marker of tumor initiating cells (Li, 2013). Studies have shown that CD133^+^ NB cells can efficiently form tumor spheres and exhibit high resistance to doxorubicin treatment by upregulating ABCG2 (Mahller et al., 2009; Zhong et al., 2018). The *FZD6* is negatively related to OS in patients with NB (Cantilena et al., 2011). Similarly, *LGR5*, a WNT-reactive G-protein-coupled receptor (GPCR) protein, was significantly associated with event-free survival (EFS) in high-risk NB subpopulations (Vieira et al., 2015). *LGR5* is specifically expressed in CSCs and is known to support the WNT/β-catenin signaling pathway as R-spondins receptors and promote tumorigenesis (Forgham et al., 2015). Elevated *LGR5* levels in IMCT-resistant cells were associated with aggressive phenotypes, and cells with high *LGR5* levels were highly resistant to chemotherapy (Forgham et al., 2015). In addition, upregulation of *ALDHs* was associated with retinoic acid (RA) tolerance (Marcato et al., 2011). Recent studies have found that surface expression of CD114, a G-CSF receptor, as a putative marker for NB-CSCs. Different from other surface markers, CD114-expressing SP < 1%, and showed a variety of CSC characteristics, its tumorigenic ability is 10 times that of CSC (Hsu et al., 2013).

As noted above, drug resistance and cancer recurrence are primarily influenced by preexisting CSCs derived from normal stem cells in specific settings. The general hypothesis is that these preexisting CSCs cause therapeutic resistance and/or disease relapse due to their unique cloning selection, self-renewal, clonal amplification, stem maintenance, and plasticity. Given the importance of targeting acquired therapeutic resistance to CSCs and treating high-risk invasive NB, some recent research has focused appropriately on developing improved treatment strategies. These strategies include targeting specific surface markers, modulating signaling pathways, modulating microenvironmental signals, inhibiting drug efflux pumps, manipulating miRNA expression, and inducing apoptosis and differentiation of CSCs (Bahmad et al., 2018).

### 3.4 Epithelial-to-mesenchymal transition

EMT is a trans-differentiation process in which epithelial cells lose intercellular contacts and apical-basal polarity and acquire mesenchymal fibroblast migration phenotype. EMT plays a key role in the formation and migration of neural crest cells, which is meaningful in neurodevelopment (Acloque et al., 2009). However, EMT is also considered to be a pathological mechanism by which tumors acquire the ability to migrate and invade, leading to disseminated disease (Thiery, 2002). EMT is an important feature in the development of therapy resistance in NB (Piskareva et al., 2015). The expression levels of EMT-related genes were different in resistant NB cells. Decreased *KRT19* expression was significantly associated with NB tumor progression, *MYCN* expansion, and poor prognosis. Similarly, decreased *ERBB3* expression was associated with *MYCN* expansion and poor survival rate (Nozato et al., 2013). The study of Naiditch et al. demonstrated that human SK-N-SH and SK-N-BE (2) C NB cells showed mesenchymal changes and transition to drug resistance through multiple pathways (Naiditch et al., 2015).

### 3.5 Autophagy

The complex roles of autophagy in therapy resistance have been excessively studied. As a pro-death or pro-survival cellular process, autophagy participates in therapy resistance in various types of cancer through a bi-directional and context-dependent way (Lei et al., 2022). In NB treatment, chemotherapy induces autophagy *in vitro* and *in vivo*. Inhibition of autophagy by hydroxychloroquine (HCQ) sensitizes NB cells to vincristine (Belounis et al., 2016). Similarly, Chen et al. (2022) reported that chemotherapeutic agents including cisplatin, cyclophosphamide, and etoposide combined with chloroquine (CQ) increased the chemotherapeutic sensitivity and cell apoptosis of high-risk NB cells. These facts indicated that autophagy is closely associated with chemoresistance and can be a potential target in the treatment of NB. Various signaling molecules or pathways contribute to the autophagy-mediated therapy resistance of NB. Wang et al. (2015) reported that High mobility group box 1 (*HMGB1*) promotes resistance of NB cells to doxorubicin, cisplatin, and etoposide by inducing Beclin-1-mediated autophagy. Additionally, inhibition of autophagy by galectin-1 knockdown sensitizes NB cells to cisplatin. Galectin-1 is a member of galectin family. This study suggested that galectin-1 is a potential target to combat chemoresistance of NB (Gao and Wang, 2019). Additionally, the resistance of NB cells to cisplatin was developed following autophagy induction. Silencing of lncRNA *SNHG7* suppressed cisplatin-induced autophagy by regulating miR-329–3p/MYO10 axis, thus decreasing cisplatin resistance. Hence, lncRNA *SNHG7* was regarded as a potential target to overcome cisplatin resistance in NB chemotherapy (Wang B et al., 2020).

### 3.6 Tumor microenvironment

It is well established that the inflammatory and immunosuppressive tumor microenvironment (TME) is involved in tumor resistance to multiple therapies (Erin et al., 2020). Treatment of NB cells with interleukin (IL)-6 rapidly activated the STAT3 to resist IL-6-induced apoptosis. Moreover, the soluble IL-6 receptor (sIL-6R) produced by human monocytes promoted the IL-6-mediated STAT3 activation. This study demonstrated an IL-6/sIL-6R/STAT3 interactive pathway which leads to environment-mediated treatment resistance (Ara et al., 2013). Extracellular vesicles (EVs), such as microvesicles and exosomes, are important components in TME. EVs are secreted by various cell types to participate in the intercellular communication. The recipient cells take up the donor cell-derived EVs which contain multiple bioactive cargoes such as microRNAs (miRNAs), long non-coding RNAs (lncRNAs), circular RNAs (circRNAs), and proteins (Mathieu et al., 2019; Huang M et al., 2021). In the TME, endothelial cells, immune cells, fibroblasts, and other cells interact with tumor cells through EVs. Liu et al. found that NB-derived small EVs promote resistance of NB cells to dinutuximab by creating an immunosuppressive TME. Inhibition of small EV secretion by tipifarnib was proposed as a novel strategy to enhance the efficacy of anti-GD2 immunotherapy in high-risk NB patients (Liu et al., 2022). A study by Kishore et al. demonstrated that exosome-mediated transfer of miR-21 and miR-155 between NB cells and monocytes confers resistance to cisplatin in NB chemotherapy. This process may be mediated by the exosomic miR-21/TLR8-NF-кB/exosomic miR-155/TERF1 signaling pathway (Challagundla et al., 2015). It is well accepted that N-Myc amplification is associated with high risk of NB (Matthay et al., 2016). Heather et al. found that exosomes derived from N-Myc-amplified NB cells mediated the resistance of non-N-Myc-amplified cells to doxorubicin-induced apoptosis. Further research revealed that exosomes from N-Myc-amplified cells may transfer aggressive phenotypes such as *TGS101, FlOT1, VPS35, NEDD4*, *β-catenin*, and *RhoA* to non-Myc-amplified cells (Colletti et al., 2017; Fonseka et al., 2019). Another study reported an exosome-based mechanism of doxorubicin resistance in NB. Tan et al. identified a highly expressed biomarker, namely, circular RNA *DLGAP4* (*circ DLGAP4*), in doxorubicin-resistant NB cells. The resistant cells deliver *circ DLGAP4* to the sensitive cells *via* exosomes. Exosome-derived *circ DLGAP4* induces doxorubicin-resistance by regulating miR-143/HK2 axis (Tan et al., 2022). In conclusion, exosomes appear to be novel targets to reverse therapy resistance.

### 3.7 Anti-disialoganglioside antibody internalization

Although the introduction of anti-GD2 mab has improved the survival outcome of high-risk NB patients, there are still some children with disease recurrence or continuous progression, which is closely related to the resistance of anti-GD2 mab (Schumacher-Kuckelkorn et al., 2017). However, the mechanism of resistance to GD2 mab immunotherapy is still poorly understood. Dr. Shahab’s team published a paper that provided new evidence on the mechanism of anti-GD2 antibody resistance, showing that the internalization of anti-GD2 mab in NB cells is an important additional factor for the development of resistance to immunotherapy (Tibbetts et al., 2022).

### 3.8 Other signaling pathways or comprehensive mechanisms

In solid tumors, therapy resistance is often acquired by the alteration of signaling pathways in the general single-agent continuous targeted therapy (Qi et al., 2015). mTOR plays an important role in cell signal transduction to regulate cancer cell growth. AZD8055, a novel and potent inhibitor of ATP-competitive and specific mTOR kinase, was verified to be an effective reagent to suppress cancer cell growth (Cirstea et al., 2014). According to previous studies, a single prolonged exposure to AZD8055 resulted in acquired resistance in NB cells. A study by Xu et al. found that the drug resistance of NB to AZD8055 was associated with the overactivation of MEK/ERK pathway (Xu et al., 2018).

Some factors mediate therapy resistance through comprehensive mechanisms. A study by Du et al. demonstrated that Cathepsin L (CTSL), which is upregulated in NB patients, was closely related to cisplatin and doxorubicin resistance of NB. CTSL facilitates chemoresistance by promoting the expression of ABCB1 and ABCG2, inhibiting the autophagy and apoptosis of NB cells. Therefore, CTSL may be a potential target to combat chemoresistance of NB to cisplatin and doxorubicin (Du et al., 2022). P2X7 receptor isoform B also contributes to NB chemoresistance through multiple mechanisms including autophagy inhibition, EMT induction, MRP-type transporter upregulation, retinoids resistance (Arnaud-Sampaio et al., 2022).

## 4 Reversal strategies for therapy resistance of NB

**FIGURE 3:**
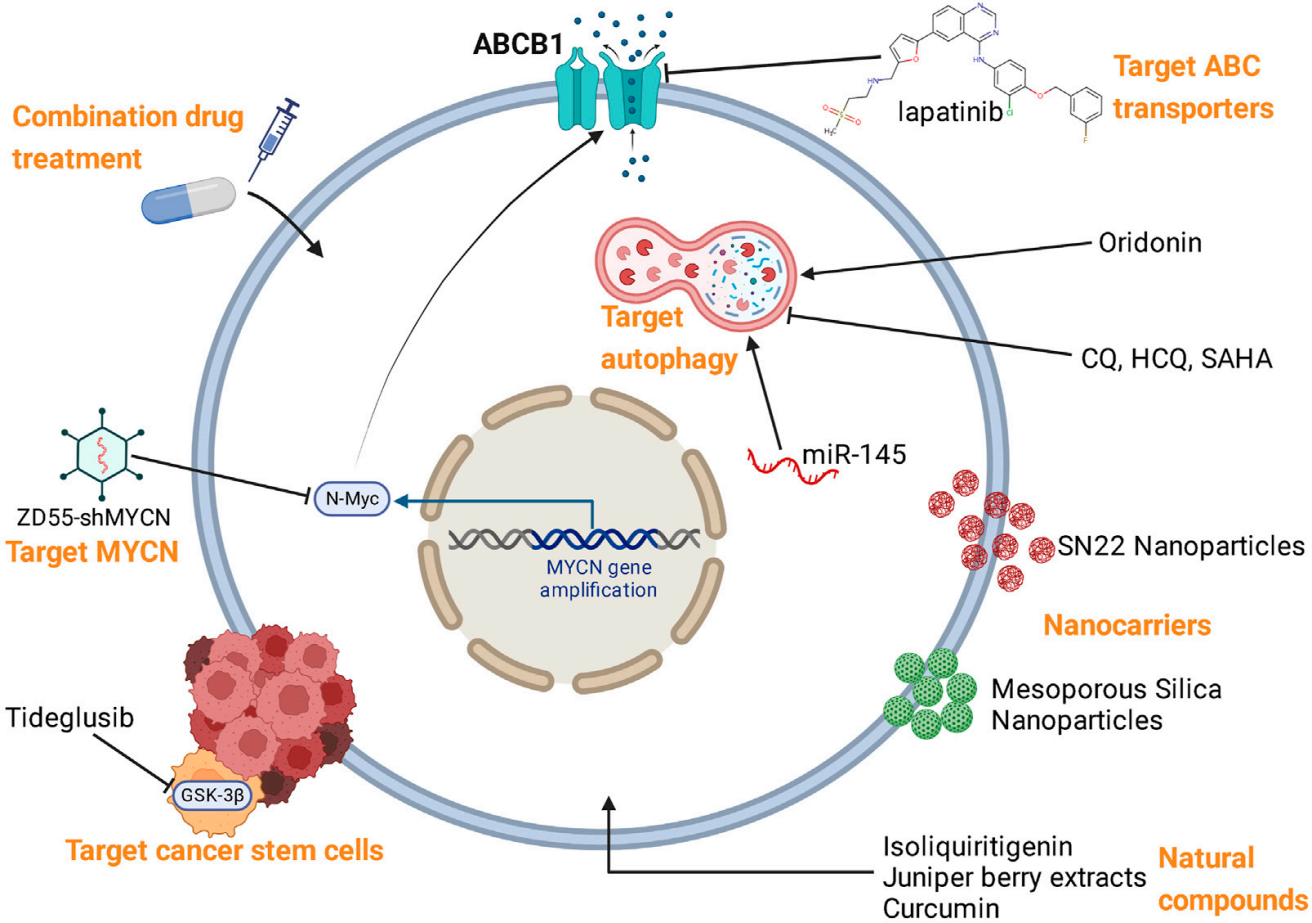
Reversal strategies for therapy resistance of NB.

Based on the mechanisms of NB resistance to multiple therapies, various reversal strategies have been proposed, which were summarized in Figure 3; Table 2.

**TABLE 2:** Reversal strategies against therapy resistance of NB.

Interventions	Sensitize NB to	Mechanism	References
GNF-4256	Irinotecan and temozolomide	TRK inhibition	Croucher et al. (2015)
AZD0156	Temozolomide + irinotecan	ATM inhibition	Koneru et al. (2021)
si-HDAC8	Doxorubicin	HDAC8 inhibition/miR-137 upregulation	Zhao et al. (2017)
arecaidine propargyl ester (APE)	Doxorubicin or cisplatin	M2 agonist/ABC efflux pumps	Luciano et al. (2020)
lapatinib	YM155	ABCB1 inhibition	Radic-Sarikas et al. (2017)
ZD55-shMYCN	Doxorubicin	Targeting MYCN	Li Y. J et al. (2017)
Tideglusib	Doxorubicin, cisplatin, carboplatin and etoposide	Targeting GSK-3β to inhibit CSC	Bahmad et al. (2021)
SN22 encapsulated in nanoparticles	Camptothecin analog SN22	Enhancing tumor cell killing efficacy	Nguyen et al. (2020), Alferiev et al. (2022)
NOX4 silencing	Chemotherapies	Suppressing PI3K/AKT signaling pathway	Yu et al. (2019)
doxorubicin-encapsulated drug delivery system with P-gp siRNA	Doxorubicin	Restoring the drug concentration at essential level	Meng et al. (2010)
a novel peptide-drug bioconjugate	Doxorubicin	Enabling the efficient intracellular Delivery	Lelle et al. (2017)
CQ	Vorinostat, panobinostat, ponatinib	Inhibiting autophagy	Corallo et al. (2020), Korholz et al. (2021)
HCQ	GX 15–070	Inhibiting autophagy	Cournoyer et al. (2019)
hydroxamic acid	Paclitaxel	Targeting TRP14-mediated autophagy	Zhen et al. (2017)
Overexpression of microRNA-145	Cisplatin, vincristine, and radiation	Enhancing autophagy	Kim et al. (2020)
Oridonin	NVP-BEZ235	Enhancing autophagy	Zhang et al. (2016)
curcumin	Doxorubicin	Upregulation of p53 and p21	Namkaew et al. (2018)

### 4.1 Combination with enzyme inhibitors or M2 receptor agonist

Some enzyme inhibitors including TRK inhibitors, ATM inhibitors, and *HDAC8* inhibitors can reverse drug resistance when combined with conventional chemotherapy drugs. GNF-4256 is a novel selective pan-TRK inhibitor identified by the Genomics Institute of the Novartis Research Foundation. Croucher et al. (2015) discovered that GNF-4256 enhanced growth inhibition of NB cells *in vitro* and *in vivo* when combined with conventional chemotherapy agents including irinotecan and temozolomide. The unrestricted proliferation of cancer cells requires maintaining telomeres (Hanahan and Weinberg, 2011). Most cancers maintain telomeres by activating telomerase (Heaphy et al., 2011). Additionally, some cancers use the alternative lengthening of telomeres (ALT) mechanism, which is more prevalent in tumors of mesenchymal and neuroepithelial origin including osteosarcoma, pancreatic neuroendocrine tumors, gliomas, and NB (Henson and Reddel, 2010). A study by Koneru et al. (2021) showed that the chemoresistance caused by ATM activation induced by telomere dysfunction in ALT NB could be reversed by the ATM inhibitor AZD0156. Histone deacetylases (*HDACs*), which can remove acetyl groups from the N-acetyllysines on histone and non-histone proteins, are considered to be important therapy targets for various cancers (Xu et al., 2012). Until now, 18 HDAC members have been identified in humans. Among them, the expression of HDAC8 is significantly increased in NB (Rettig et al., 2015). Among the *HDAC8*-regulated miRNAs, miR-137 has attracted much attention due to its critical role in NB progression. It has been reported that *HDAC8* can regulate the expression of miR-137, which in turn regulates the chemoresistance of cancer cells (Mahmoudi and Cairns, 2017). Studies have shown that targeted inhibition of *HDAC8* can suppress the growth of NB cells and increase doxorubicin sensitivity *via* upregulation of miR-137 and suppression of MDR1 (Zhao et al., 2017).

In recent years, the significance of muscarinic receptors has been widely reported as a new therapeutic target for the treatment of different forms of cancers. Acetylcholine (ACh) is one of the major neurotransmitters in the nervous system, which can be synthesized in different types of tumor cells. Production of ACh by tumor cells and subsequent interactions with muscarinic receptors often activate autocrine/paracrine cycles that regulate cell proliferation, migration, and angiogenesis. Study has shown that the combination of low-dose conventional chemotherapy drugs and M2 agonists can affect the resistance of NB through the decreased expression of MDR pumps (Luciano et al., 2020).

### 4.2 Modulation of ABC transporters

MDR, frequently mediated by ABC transporters, is one of the most recognized obstacles in the success of pharmacological anticancer approaches. The efflux of diverse drugs to extracellular environment is mediated by these transporters, causing insensitivity of tumor cells to chemotherapy and MDR (Feyzizadeh et al., 2022). ABC transporters, as transporters of phospholipids, lipophilic drugs, cholesterol, and other small molecules on the cell membrane, are mainly responsible for the distribution, absorption, and efflux of various compounds. Given that overexpression of ABC transporters is one of the most common mechanisms leading to drug resistance in cancer cells, inhibition of these transporters is considered an effective way to sensitize drug-resistant cancer cells (Porro et al., 2010). The strategies of ABC transporter block include regulation of protein expression and small molecular inhibitors. Using a systematic drug combination screen, Radic et al. observed a strong synergy between the EGFR kinase inhibitor lapatinib and the anticancer compound YM155 in NB cells, which is retained in several NB variants. Mechanistically, this synergistic effect is based on lapatinib-induced inhibition of the MDR efflux transporter ABCB1, which is frequently expressed in drug-resistant NB cells, thereby prolonging and enhancing the cytotoxicity of YM155 (Radic-Sarikas et al., 2017).

### 4.3 Targeting MYCN by oncolytic virus with shRNA

The MDR-associated protein (MRP) genes encoding transmembrane glycoproteins are key regulators of MDR. The expression of MRP in NB is closely related to the MYCN oncogene. A MYCN-dependent oncogenic pathway plays a vital role in promoting the aggressive, intrinsically resistant disease phenotype (Molenaar et al., 2009). It has been reported that the expression level of MYCN and MRP is higher in doxorubicin-resistant cells than in parental cells. Silencing the MYCN gene in doxorubicin-resistant cells downregulates MRP and resensitizes these resistant cells to doxorubicin. Downregulation of MYCN and MRP increased intracellular doxorubicin levels and enhanced the therapeutic effect of doxorubicin (Haber et al., 1999). A novel type of oncolytic virus with shRNA, ZD55-shMYCN, can reverse MDR in NB by targeting MYCN to inhibit tumor cell proliferation and induce NB cell apoptosis (Li Y et al., 2017).

### 4.4 Targeting the cancer stem cells

Due to the characteristics of self-renewal, pluripotency, unlimited proliferation, angiogenesis, and immune evasion, CSCs cannot be completely eliminated by traditional treatment (Bahmad et al., 2019). These resistant cells lead to tumor recurrence due to the expression of DNA repair mechanisms, detoxification enzymes, anti-apoptotic proteins, and MDR transporters (Ross and Spengler, 2007). Therefore, CSCs play an important role in NB progression, recurrence, and poor prognosis (Aravindan et al., 2019). Glycogen synthase kinase 3β (GSK-3β) is an active proline-directed serine/threonine kinase, which is closely related to tumor formation and progression. The study by Bahmad et al. evaluated the anti-tumor effect of Tideglusib, an irreversible inhibitor of GSK-3β, on NB CSCs *in vitro* and *in vivo*. Their findings showed that Tideglusib could be used as an effective drug targeting NB CSC, thereby overcoming treatment resistance (Bahmad et al., 2021). Although many promising CSC surface markers have been identified, there are two key factors: 1) the lack of specific candidates that are universally available among all NB-CSCs in tumors; 2) The presence of candidate markers in normal non-tumorigenic stem cells highly limits their use in developing csc targeting methods (Aravindan et al., 2019).

### 4.5 Nanocarriers in resistance reversal

Nanocarriers are excellent platforms to enhance the accumulation of chemotherapy drugs in tumor cells. Combining nanotechnology with existing therapies such as gene therapy and P-gp inhibitors has been shown to improve drug resistance. Nanoparticles, when carefully designed, have the potential to control drug release profiles to achieve optimal cytotoxicity and avoid drug resistance by sensitizing cancer cells to chemotherapy (Palakurthi et al., 2012). SN22 is a topoisomerase I inhibitor that has shown potent anticancer activity in early preclinical studies, but its clinical application is limited due to its lack of solubility in standard delivery vehicles. A recent study modified the structure of the camptothecin analog SN22 and reversibly coupled it with a redox-free tocol derivative (tocopheryl oxalate) to convert it to a PEGylated sub-100 optimal stable encapsulation. Controlled release in nanometer nanoparticles (NPs) enhance their pharmacological selectivity, favorably alter biodistribution, enhance tumor cell killing efficacy, and overcome drug resistance (Nguyen et al., 2020; Alferiev et al., 2022).

### 4.6 Reversal of hypoxia-induced resistance

Hypoxia is the main cause of treatment failure in various types of malignant tumors including NB (Ward et al., 2013). In hypoxic environments, cancer cells acquire hypoxic resistance through genetic and adaptive changes to survive and proliferate (Li et al., 2016). Hypoxia-inducible factor 1α (HIF-1α), an oxygen-dependent activator of transcription, improve the viability of hypoxic cells to participate in tumor angiogenesis and mammalian development (Lee et al., 2004; Zhang et al., 2015). Hypoxia induces a high rate of glycolysis in tumors. Therefore, inhibiting glycolysis to reduce cancer cell viability in hypoxic environments helps to attenuate the hypoxic resistance of cancer cells (Cao et al., 2007). NOX4 was found to be highly expressed in human NB cells under the condition of hypoxia (Liu et al., 2017). Studies have shown that silencing NOX4 inhibits hypoxia-induced glycolysis and cell growth by suppressing PI3K/AKT signaling pathway, which would attenuate hypoxia resistance. As a result, the tumor progression and drug resistance of NB is restrained (Yu et al., 2019).

### 4.7 Combination therapy *via* bioconjugates

Bioconjugation usually involves two molecules linked by covalent bonds, at least one of which should be a biological source or biomolecule (Antonio et al., 2019). In cancer therapeutic drugs, these biological molecules used for conjugation are mainly ligands targeting tumor-specific antigens (Varshosaz et al., 2016). It can also be peptide (Szweda et al., 2016), glycoprotein (Casanas Pimentel et al., 2016), aptamer, or interferon with anticancer properties (Wadhawan et al., 2019). The unique advantage of bioconjugates is that they can selectively deliver therapeutic drugs to pathological sites and increase the retention of molecules in the blood circulation system. Their delivery mechanism is based on active drug delivery (Sun et al., 2012). Researchers have synthesized bioconjugates to overcome MDR by conjugating certain molecules. Meng et al. (2010) conjugated a doxorubicin-encapsulated drug delivery system with P-gp siRNA that knocked down the P-gp gene expression. This system restored the drug concentration at essential level to induce apoptosis of cancer cells. Marco et al. described the synthesis and characteristic of a novel bioconjugate, consisting of an octaarginine cell-penetrating peptide and a highly DNA-affine doxorubicin dimer. Their study showed that the novel bioconjugate could successfully overcome the drug resistance of NB cells (Lelle et al., 2017). The therapeutic effect of bioconjugation becomes more significant compared with their individual effects. However, the current application of bioconjugates faces two major challenges. First, the efficacy of *in vitro* test often cannot be translated into clinical staging. Another challenge is the cost of production. When combined with therapeutic biomolecules such as antibodies, aptamers, nucleic acids, and peptides, the production cost will increase more or less.

### 4.8 Interference with autophagy

Autophagy is a ubiquitous cellular self-degradation process that involves the degradation and recycling of cytosolic components through the lysosomal pathway. Autophagy is closely related to therapy resistance of NB. Accumulating evidence indicated that autophagy inhibition significantly reduced chemoresistance of NB (Belounis et al., 2016). Korholz et al. (2021) investigated the impact of broad-spectrum HDACIs on autophagic flux. The results showed that autophagy induced by vorinostat and panobinostat transcriptionally upregulated autophagy-related genes in NB cells and induced nuclear translocation of the transcription factors FOXO1 and FOXO3a. Combination of vorinostat or panobinostat with the autophagy modulating agent CQ enhanced NB cell death. Ponatinib (PON) is a tyrosine kinase inhibitor for the treatment of NB. The cytotoxicity of PON is commonly impaired by autophagy, indicating the cytoprotective role of autophagy in response to PON. A study revealed that inhibition of autophagy by CQ significantly ameliorates the PON resistance *in vitro* and *in vivo*, making combination of CQ and PON a promising strategy for NB treatment (Corallo et al., 2020). Similarly, combining HCQ with GX 15–070, a Bcl-2 family proteins inhibitor, exerts synergistic effect and increases chemosensitivity in NB (Cournoyer et al., 2019). Suberoylanilide hydroxamic acid (SAHA) is an inhibitor of thioredoxin-related protein 14 (TRP14) which was upregulated in NB cells after paclitaxel treatment. Zhen et al. (2017) found that SAHA sensitizes NB cells to paclitaxel by targeting TRP14-mediated autophagy. In addition to the cyto-protective role, the pro-death role of autophagy for cancer cells has also been demonstrated. Overexpression of microRNA-145 sensitizes Cisplatin-resistant, Vincristine-resistant, and radiation-resistant NB cells to their corresponding treatment by enhancing autophagy (Kim et al., 2020). Oridonin, a natural biologically active compound, sensitizes NB cells to NVP-BEZ235 *in vitro* and *in vivo* through enhancing autophagy (Zhang et al., 2016). In conclusion, targeting autophagy is a promising approach to overcome therapeutic resistance in NB.

### 4.9 Natural compounds

Plant-derived natural products with pleiotropy and low toxicity have been introduced as ideal candidate agents to develop promising MDR modulators. Significantly, several natural substances are effective against NB cells *in vitro*. Flavonoids and polyphenols are known as promising therapeutic compounds (Dobrzynska et al., 2020). Isoliquiritigenin, a natural extract of flavonoid, induces necrotic cell death and cell cycle arrest by down-regulating ATP in NB cells (Escobar et al., 2019). In addition, juniper berry extracts containing flavonoids, upregulate p53 and induce endoplasmic reticulum stress and apoptosis in NB cells (Lantto et al., 2016). Furthermore, curcumin, a natural polyphenolic compound derived from the South Asian turmeric plant (Curcuma longa), displays good anticancer properties by inhibiting the serine-threonine kinases AKT/NF-κB signaling. It can induce mitochondrial dysfunction and p53 upregulation, finally leading to apoptosis (Zhai et al., 2020). Another potential application of curcumin in the treatment of NB is in combination with conventional chemotherapeutic agents, especially cisplatin and doxorubicin (Namkaew et al., 2018). Curcumin also demonstrates synergistic effects with other natural substances and radiation therapy against NB cells (Farhood et al., 2019). Combining curcumin with lower doses of chemotherapeutic drugs and radiation can achieve high antitumor efficacy, as well as low toxicity and drug resistance. Moreover, although curcumin has the potential to slow NB progression and fight cancer drug resistance, its clinical viability is limited due to its poor oral bioavailability, low water solubility, and rapid metabolism. A number of novel curcumin formulations, including nanoparticles, lipid carriers, nanosuspensions, and microemulsions have been proposed to overcome these problems (Sadegh Malvajerd et al., 2019).

## 5 Conclusion and perspectives

Currently, different treatments against NB have displayed favorable clinical outcomes such as chemotherapy, radiotherapy, targeted therapy, and immunotherapy. However, the genetic heterogeneity of NB limits the efficacy of existing treatment modalities. Many patients do not respond to treatments since NB develops resistance through complex mechanisms. It is reported that almost 50% of patients developed resistance to anti-GD2 treatment and relapsed (Chan and Chan, 2022). Hence, there is an urgent need to understand the resistant mechanism and explore reversal strategies. As mentioned above, ABC transporters, miRNAs, CSCs, EMT, autophagy, TME, and some signaling pathways may be potential targets to combat therapy resistance of NB. Recent studies have identified a large number of genetic alterations and dysfunctional pathways related to NB resistance through high-throughput “omics” techniques. Continued efforts should be taken to discover novel therapeutic targets against NB resistance and prognostic markers which can access patients’ response to treatment. At present, some monotherapies or combined therapies targeting NB resistance are being tested in preclinical or clinical studies. For instance, chemotherapy in combination with miR-based treatment was regarded as a promising method for clinical management of progressive NB. Novel strategies have been proposed and are under investigation such as inducing pyroptosis in apoptosis resistant-NB cells (Wang et al., 2022). In conclusion, this review demonstrated the complex mechanisms (Figure 2) and various reversal strategies (Figure 3; Table 2) of NB resistance to multiple treatments. Further exploration is needed to overcome the therapy resistance of NB and improve therapy efficacy. We expect that patients will benefit from the novel interventions targeting NB resistance in the future.
